# Emerging Therapeutic Targets and Experimental Drugs for the Treatment of NAFLD

**DOI:** 10.3390/diseases6030083

**Published:** 2018-09-19

**Authors:** Pratima Dibba, Andrew A. Li, Brandon J. Perumpail, Nimy John, Sandy Sallam, Neha D. Shah, Waiyee Kwong, George Cholankeril, Donghee Kim, Aijaz Ahmed

**Affiliations:** 1 Division of Gastroenterology, Women & Infants Hospital/Warren Alpert School of Medicine, Brown University, Providence, RI 02905, USA; pratima_dibba@brown.edu; 2 Department of Medicine, Stanford University School of Medicine, Stanford, CA 94304, USA; andrewli@stanford.edu; 3 Drexel University College of Medicine, Philadelphia, PA 19129, USA; brandonperumpail@gmail.com; 4 Division of Gastroenterology and Hepatology, Stanford University School of Medicine, Stanford, CA 94304, USA; nionnj@gmail.com (N.J.); SSallam@stanfordhealthcare.org (S.S.); NeShah@stanfordhealthcare.org (N.D.S.); WKwong@stanfordhealthcare.org (W.K.); georgetc@stanford.edu (G.C.); dhkimmd@stanford.edu (D.K.)

**Keywords:** NAFLD, NAFLD, NASH, nonalcoholic fatty liver disease, drug therapy, chemokine receptor 2 and 5, galectin-3 protein, toll-like receptor 4, fibroblast growth factor 19, selective thyroid hormone receptor-beta, apoptosis signal-regulating kinase 1, acetyl-coenzyme A carboxylase, farnesoid X receptor, inflammasomes

## Abstract

The two main subsets of nonalcoholic fatty liver disease (NAFLD) include: (1) nonalcoholic fatty liver (NAFL), the more common and non-progressive subtype; and (2) nonalcoholic steatohepatitis (NASH), the less common subtype, which has the potential to progress to advanced liver damage. Current treatment strategies have focused on lifestyle management of modifiable risk factors, namely weight, and on the optimization of the management of individual components of metabolic syndrome. Various hypothetical pathogenic mechanisms have been proposed, leading to the development of novel drugs with the potential to effectively treat patients with NASH. Numerous clinical trials are ongoing, utilizing these experimental drugs and molecules targeting specific mechanistic pathway(s) to effectively treat NASH. Some of these mechanistic pathways targeted by experimental pharmacologic agents include chemokine receptor 2 and 5 antagonism, inhibition of galectin-3 protein, antagonism of toll-like receptor 4, variation of fibroblast growth factor 19, agonism of selective thyroid hormone receptor-beta, inhibition of apoptosis signal-regulating kinase 1, inhibition of acetyl-coenzyme A carboxylase, agonism of farnesoid X receptor, antibodies against lysl oxidase-like-2, and inhibition of inflammasomes. Emerging data are promising and further updates from ongoing clinical trials are eagerly awaited.

## 1. Introduction

The two subsets of nonalcoholic fatty liver disease (NAFLD) include nonalcoholic fatty liver (NAFL), the more common and non-progressive subtype, and nonalcoholic steatohepatitis (NASH), the less common subtype, which has potential to progress to advanced liver damage. Current treatment strategies have focused on lifestyle management of modifiable risk factors (namely weight) and on the optimization of the management of individual components of metabolic syndrome [1,2,3,4]. The European Association for the Study of the Liver (EASL), the European Association for the Study of Diabetes (EASD), the European Association for the Study of Obesity (EASO), the Asia-Pacific Working Party, and the American Association for the Study of Liver Disease (AASLD) have commented on other therapeutic interventions, particularly oral hypoglycemic agents (otherwise known as insulin-sensitizing drugs), vitamin E, lipid-lowering agents, and bariatric surgery [1,2,3,4]. Various key mechanistic pathways involved in the pathogenesis of NAFLD are the focus of targeted therapies to treat patients with NASH, the subset of NAFLD with most the rapidly progressive liver damage. Some of these most promising experimental pharmacologic agents include chemokine receptor 2 (CCK2) and 5 (CCK5) antagonism, inhibition of galectin-3 protein, antagonism of toll-like receptor 4 (TLR4), variation of fibroblast growth factor 19 (FGF19), agonism of selective thyroid hormone receptor-beta, peroxisome proliferator-activated receptor (PPAR) agonism, inhibition of apoptosis signal-regulating kinase 1, inhibition of acetyl-coenzyme A carboxylase, agonism of farnesoid X receptor (FXR), antibodies against lysl oxidase-like-2 (LOXL2), and inhibition of inflammasomes [5,6,7,8,9,10,11]. The focus of this review article is to discuss the experimental drugs with promising emerging data in patients with NASH. 

## 2. Pathophysiology and Pathogenesis of NAFLD

Briefly, NAFLD is the accumulation of fat within the hepatocytes when import or synthesis of fat exceeds its export or degradation [12]. Subsequently, a cascade of events occur in these lipotoxic hepatocytes, including activation of immune mediators and inflammation, matrix remodeling via fibrogenesis and fibrinolysis, angiogenesis, and mobilization of liver progenitor cells [12]. With the recent advances in the field of drug development, there has been an increasing focus on modulating the mediators of these pathways as the therapeutic target in patients with NASH. Other potential drug targets and proposed pathogenic mechanisms that are being investigated include inhibition of caspases, the adenosine system, and cannabinoid receptor and enzyme modulation [13].

## 3. Treatment Guidelines

Due to the variety of proposed mechanisms for NAFLD and ambiguity in exact pathogenesis, recommendations proposed by the EASL, EASD, EASO, the Asia-Pacific Working Party, and the AASLD focus primarily on lifestyle modifications, without clear guidance on pharmacotherapy or surgical options [1,2,3]. Specifically, they advise that patients with NAFLD, excluding those with NASH, should achieve weight loss through a combination of dietary modification and exercise [1,2,3,4]. There was insufficient data to recommend for or against oral hypoglycemics (i.e., metformin, thiazolidinediones), vitamin E, lipid-lowering agents, or bariatric surgery [1]. In a more recently published “guidance” document as an update to the 2012 guidelines, the AASLD additionally recommends pharmacologic treatment primarily in patients with biopsy-proven NASH with fibrosis and makes mention of two drugs being tested in phase 3 registration trials, obeticholic acid (OCA) and elafibranor [3,4].

## 4. Potential Drug Targets for the Treatment of NAFLD

### 4.1. Obeticholic Acid and GS-9674—Farnesoid X Receptor Agonists

FXR is a hormone receptor expressed in the liver that functions in the regulation of bile acid [14]. Furthermore, FXR plays a key role as a mediator of inflammatory responses, in the regulation of lipid-related pathways, and in glucose metabolism. Therefore, it has been implicated in pathogenesis of NAFLD [15]. Its deficiency in murine models with induced NASH [16] and its agonism demonstrated reduction in insulin resistance and hepatic steatosis [17]. OCA is an FXR agonist that was tested in a double-blind, placebo-controlled, proof of concept study (NCT00501592) to evaluate its effect on insulin sensitivity in patients with type 2 diabetes and NASH [17]. Results demonstrated reductions in hepatic inflammation and fibrosis, and an increase in insulin sensitivity [17]. OCA was also tested in 283 patients in a multicenter, double-blind, placebo controlled, parallel group, randomized phase 2b clinical trial (FLINT; NCT01265498) whose primary outcome was an improvement in centrally scored liver histology as defined by improvement in the NAFLD activity score (NAS), which is defined as a validated histologic feature scoring system designed by the Pathology Committee of the NASH Clinical Research Network to encompass the entire spectrum of lesions of NASH for use in clinical trials [18]. In those treated with OCA, NAS improved by at least two points without worsening of fibrosis as assessed by biopsies [19]. Although decisions were made against biopsies at 24 weeks, improvement of alanine aminotransaminase supported the decision to proceed with treatment to 72 weeks of treatment [19]. At 72 weeks, 45% of patients receiving OCA demonstrated improved liver histology compared with 21% of patients in the placebo group [19]. Other benefits observed include weight loss in those with NASH, with favorable effects on alkaline phosphatase, lipids, and blood glucose [20]. OCA is now in a phase 3 trial (REGENERATE; NCT02548351) to evaluate its effect on liver histology and outcomes in over 2000 patients with biopsy-proven NASH with fibrosis in stages 2–3 [21]. More recently, a phase 3 clinical trial (REVERSE; NCT03439254) studying OCA in compensated cirrhosis was initiated [22]. Unlike OCA, an FXR agonist, GS-9674, is still in the preliminary phases of clinical evaluation [14]. GS-9674 produces FGF19, a protective hormone with reduction in its levels in patients with NASH, insulin resistance, and metabolic syndrome; and reversal to normal levels following bariatric surgery [14]. In murine studies, rodents were fed with a fast-food diet to simulate NASH and then treated with GS-9674; the results were notable for a significant reduction in aminotransferases, hepatic steatosis, and fibrosis [14]. GS-9674 increased the FGF19 levels and was noted to have a favorable safety profile in a randomized, double-blind, placebo-controlled study of healthy volunteers, confirming its biologic activity and potential in patients with NASH [23]. Its safety is being evaluated in a phase 2 trial of 140 patients (NCT02854605) [24].

### 4.2. Elafibranor: A Dual Peroxisome Proliferator-Activated Receptor (PPAR) Alpha/Delta Agonist

Peroxisome proliferator-activated receptor alpha (PPARα) is a transcription factor that works with PPARδ and has been found to improve hepatic steatosis, inflammation, and fibrosis in pre-clinical models of NASH [25]. It has been implicated in the pathogenesis of NAFLD/NASH and has been a target for pharmacotherapy in the treatment of NASH [25]. Elafibranor, a dual PPAR α/δ agonist, was evaluated in a phase 2 trial (GOLDEN-505, NCT01694849) over 52 weeks in a sample size of 276 patients [26,27]. The primary outcome measured was reversal without worsening of hepatic fibrosis, and secondary outcomes measured changes in NAS, improvements in histologic scores of steatosis, ballooning, inflammation and fibrosis, improvement in liver enzymes, lipid and glycemic parameters, insulin resistance, and inflammatory markers, while measuring safety and tolerability [26]. In higher doses, elafibranor was superior to the placebo in demonstrating reversal of NASH without worsening of fibrosis [26]. There were also reductions in hepatic fibrosis, hepatocyte ballooning, and NAS alongside improvements in liver transaminases [26]. Improvements in cardiometabolic parameters were observed, particularly triglycerides, low-density lipoprotein, high-density lipoprotein, fasting serum glucose in diabetics, fasting insulin, homeostatic model assessment of insulin resistance (a method quantifying insulin resistance), and circulating free fatty acids [26]. C-reactive protein, fibrinogen, and haptoglobin were also reduced in the treatment group [26]. Although it was generally safe and well-tolerated, patients treated with elafibrinor demonstrated statistically significant increases in creatinine [3,26,28]. Elafibranor is now in a phase 3 trial (RESOLVE-IT, NCT02704403) [4,29].

### 4.3. Cencriviroc: CCR2/CCR5 Dual Antagonist

The CCR2 and CCR5 dual antagonist, cenicriviroc (CVC), currently undergoing evaluation in patients with human immunodeficiency virus (HIV) [30] and NASH [5,31]. The ligand of CCR2, C-C chemokine ligand 2 (CCL2) is secreted by Kupffer cells when hepatocytes are injured, causing recruitment of monocytes in the liver and maturation of monocytes into macrophages [31]. The macrophages then secrete cytokines, which activate hepatic stellate cells that stimulate collagen and promote hepatic fibrosis [31]. CCR2 and CCR5 promote activation and migration of Kupffer cells and hepatic stellate cells and increase inflammatory cells [31]. CCR2 and CCR5 were identified as mediators in hepatic fibrogenesis and NASH in animal (rat) models [32,33,34]. The antagonism of CCR2 and CCR5 by CVC was evaluated in thioacetamide-induced rat models of liver fibrosis and mouse models of diet-induced NASH [33]. CVC demonstrated reduction in collagen deposition, collagen type 1 proteins, and mRNA in fibrosis [33]. In the NASH model, there was also a reduction in NAS [33]. Based on studies in HIV, CVC was found to have a favorable safety profile and was well-tolerated in patients with mild to moderate hepatic impairment, resulting in a fast track designation by the United States Food and Drug Administration (FDA) [31]. CVC was evaluated by Friedman et al. in a phase 2b, randomized, double-blind multinational trial (CENTAUR study, NCT02217475) at 150 mg over two years in a total of 289 enrolled patients [31]. The primary endpoint of the study, which was developed based phase 2b studies of pioglitazone, vitamin E, and OCA in NASH [19,35], was hepatic histologic improvement in NAS at one year relative to the screening biopsy, as graded by an improvement of NAS by at least two points with a greater than one point reduction in lobular inflammation or hepatocellular ballooning and no worsening of fibrosis [19,31,35]. Secondary endpoints included complete resolution of steatohepatitis with no worsening fibrosis and improvement of fibrosis by more than one stage with no worsening of steatohepatitis [5,31]. At one year, although the primary endpoint was not met, study subjects experienced a ≥2-point improvement of NAS with no progression in hepatic fibrosis [5]. Additional observations include resolution of steatohepatitis with no progression of fibrosis, improvement in fibrosis by ≥1 stage (according to the NASH Clinical Research Network and Ishak staging systems), and no worsening of steatohepatitis observed in twice as many patients as those receiving the placebo [5]. There was an improvement in the intent-to-treat population although the fibrosis endpoint was met in more subjects receiving CVC versus placebo [5]. There was also a reduction in biomarkers of systemic inflammation such as C-reactive protein and interleukin-6, as well as fibrinogen [5]. Treatment benefits were greater in those with higher disease activity [5]. CVC has also demonstrated reduction in fibrosis despite ongoing steatohepatitis in ongoing murine models, although several limitations exist in these studies [36]. There were no significant differences in body weight, BMI, or fasting metabolic parameters, suggesting that perhaps drug therapy should target inflammatory and fibrotic mechanisms instead of the previously hypothesized metabolic risk factors [5]. CVC was well tolerated, with mild to moderate adverse effects noted [5]. It is currently being evaluated in a phase 3 clinical trial (AURORA; NCT03028740) due to promising findings in prior phases [37,38]. Current issues being investigated include durability of responses, divergent effects in NASH versus fibrosis, and potential long-term outcomes [37,38].

### 4.4. GR-MD-02: Galectin-3 Protein Inhibitor

Galectin-3 protein is a lectin protein which mediates the development of hepatic fibrosis and has been noted to have increased levels in macrophages surrounding lipid-laden hepatocytes in NASH [39]. In rodent models, there is conflicting evidence. One study demonstrated that galectin-3 knockout mice were resistant to hepatic fat accumulation, inflammation and fibrosis when fed a high fat diet [40]. Another study showed that galectin-null mice may be at increased risk of developing NASH although they experience decreased liver cell injury and fibrosis [40]. Traber et al. demonstrated that inhibition of galectin-3 protein via GR-MD-02, a complex carbohydrate derived from plants that bind to galectin-3, resulted in improvement in histopathologic changes of NASH-related hepatic fibrosis in rodent models, in whom fibrosis was induced by thioacetamide [40,41,42]. Based on these findings, GR-MD-02 also received fast track designation by the FDA in the treatment of NASH with advanced fibrosis [40]. In its phase 1 clinical trial versus placebo therapy, 30 subjects completed the trial; findings met the primary endpoint of favorable safety and pharmacokinetics [40]. Its safety and efficacy for the treatment of hepatic fibrosis and resultant portal hypertension is being evaluated in clinical trial NASH-CX, a one-year multicenter, parallel group, randomized, double-blinded, placebo-controlled trial of GR-MD-02 with 162 subjects (NCT02462967) [43,44]. Although the primary endpoint of this trial is to evaluate its efficacy in reducing the hepatic venous pressure gradient compared to placebo, secondary outcomes are being investigated, which include change in Ishak histopathologic staging fibrosis, base like-adjusted mean change in liver stiffness and metabolic capacity of liver, and difference in progression to cirrhosis between the two groups [43]. In a late-breaking presentation of the most recent findings of the NASH-CX trial, patients received 26 doses of the drug versus placebo over 54 weeks to evaluate its safety and efficacy in the setting of well-compensated NASH cirrhosis [44]. GR-MD-02 demonstrated a favorable trend towards improvement in NAS, although findings were not statistically significant [44]. Other findings include improvement in hepatocyte ballooning and reduction in liver cell death correlating with response to therapy, reduction in development of new varices and a favorable safety profile [44]. Based on the promising results produced in NASH-CX, GR-MD-02 proceeded to phase 3 as of May 2018 [45].

### 4.5. NGM282: Variant of FGF19

FGF19 is a gastrointestinal growth hormone that is stimulated by FXR [46]. It primarily functions in bile acid regulation, but additionally functions in glycogen synthesis and gluconeogenesis, and has also been implicated in the pathogenesis of NASH [6,46]. In FGF19 transgenic mice, decreased acetyl coenzyme A carboxylase 2 resulted in reduction of liver triglyceride levels and increase in energy expenditure [47]. One study demonstrated that FGF19 correlated with its receptor EpCAM as a marker of hepatic cancer stem cells within the fatty liver–steatosis–cirrhosis–hepatocellular carcinoma sequence through comparison of histologic stages of tissue within the sequence versus healthy hepatic tissue [48]. In rodent models, FGF19 demonstrated amelioration of hepatosteatosis by reducing fatty acid synthesis and increased fatty acid oxidation [49]. In rodent studies by Zhou et al., FGF19 was found to reduce hepatic triglyceride levels with reduction in intrahepatic cholesterol content and lipotoxic free cholesterol in diet-induced mouse models of NASH [49]. In the same study, there were reductions in aminotransaminases and improvements in histologic changes associated with NASH [49]. Studies conducted by Luo et al. in diet-fed mice models of NASH demonstrated decreased body weight, liver weight, and liver-to-body weight ratio, reflecting a decrease in total liver fat content, with improvement of steatosis, lobular inflammation, and hepatocyte ballooning [50]. Reductions in alanine transaminase, glucose, and triglycerides were also observed [50]. Because FGF19 is decreased in NASH, its dysregulated expression may be a culprit in the development of NASH [6,49]. An FGF19 analogue was studied thereafter [6]. In a clinical trial (NCT02443116), a 12-week phase 2 randomized, placebo-controlled trial of 82 patients [6,51], NGM282 (a variant of FGF19) demonstrated favorable endpoints with an overall favorable safety profile, exclusive of three serious adverse events (i.e., pleurisy, chest tightness and cardiac arrest) [52]. Namely, NGM282 demonstrated reductions in liver fat content based on magnetic resonance imaging–proton density fat fraction, decreases in biomarkers (aminotransferases, PRO-C3, Enhanced Liver Fibrosis Test), decreases in NAS, steatosis, inflammation, and ballooning, and demonstrated unprecedented anti-fibrotic activity with favorable safety and tolerability [6,53]. Adverse events that were reported include injection site reactions, diarrhea, abdominal pain, and nausea [6]. It is currently undergoing a phase 2 expansion study and will undergo a phase 2b study later this year with a primary endpoint of change in hepatic fat content [53].

### 4.6. MGL-3196: Selective Thyroid Hormone Receptor-Beta Agonist

Thyroid hormone receptor-beta agonist activity has demonstrated reductions in low-density lipoprotein, triglycerides and hepatic steatosis in humans [7,54,55]. It has demonstrated a reduction in liver fat via breakdown of fatty acids and stimulation of mitochondrial biogenesis to reduce lipotoxicity and improve hepatic function [7,54]. MGL-3196, a selective thyroid hormone receptor-beta agonist, has been targeted based on a hypothesis that thyroid hormone receptor activity may have an important role in the pathogenesis of NASH [55]. In studies performed to assess its role in dyslipidemia and hypercholesterolemia, MGL-3196 demonstrated reductions in low density lipoprotein and triglycerides, with a favorable safety profile and tolerability [55,56]. MGL-3196 was then studied in a randomized, double-blind placebo-controlled phase 2 trial (NDT02912260) in 78 study subjects with a primary endpoint of change from baseline hepatic fat fraction [7,54,57]. It demonstrated reduction of liver fat, fibrosis, and aminotransaminases at 12 weeks [7,54]. It was well tolerated, with most adverse events being classified as mild or moderate [7,54]. More recent results at the 36-week point revealed reduction in hepatic steatosis, decline in low-density lipoprotein, and decrease in aminotransaminases and improvement in hepatic fibrosis biomarkers [58]. Patients treated with MGL-3196 demonstrated a greater than 30% fat reduction at week 12, with a higher percentage of NAS reduction and NASH resolution [59]. In those with NASH resolution, fibrosis resolved in 50% of patients [59]. There is speculation that MGL-3196 will enter phase 3 trials [59].

### 4.7. Selonsertib: Apoptosis Signal-Regulating Kinase 1 Inhibitor

The inhibition of apoptosis signal-regulating kinase 1 (ASK1), which is a serine/threonine kinase, demonstrated improvement in inflammation and fibrosis in animal models of NASH [8]. In preclinical models of NASH involving mice and nonhuman primates, deletion or pharmacologic inhibition of ASK1 ultimately resulted in reduced hepatic steatosis, inflammation, and fibrosis [60,61]. Selonsertib, previously known as GS-4997, is a selective inhibitor of ASK1, which, in animal models fed with a diet high in fat, cholesterol, and sugar, reduced hepatic steatosis, fibrosis, and insulin resistance [62]. ASK1 is activated by oxidative stress and promotes hepatocellular apoptosis, inflammation, and fibrosis [8]. In a multicenter, phase 2 clinical trial (NCT02466516) with a total of 72 patients, selonsertib was tested alone versus in combination with simtuzumab (a lysl oxidase-like-2 antibody to be discussed later) [63]. Based on liver biopsies and advanced magnetic resonance imaging methods, patients treated with selonsertib had higher rates of improvement in fibrosis versus those treated with simtuzumab over a 24-week treatment period [8]. Patients with NASH and stage 2 or 3 fibrosis who responded to solensertib demonstrated reductions in hepatic collagen content, liver stiffness, alpha-smooth muscle actin, and serum markers of apoptosis (cytokeratin-18 M30 and M65) [8]. In addition, these patients also demonstrated improvements in lobular inflammation and hepatic steatosis by morphometry [8]. In another study assessing patient-reported outcomes in 70 patients with NASH and stage 2–3 fibrosis treated with selonsertib, selonsertib with simtuzumab, or simtuzumab alone, patient-reported outcomes were increased in those patients treated with selonsertib showing reduction in hepatic collagen [64]. Currently, selonsertib is undergoing evaluation in phase 3 clinical trials studying its efficacy in patients with: (1) NASH and bridging fibrosis (STELLAR-3; NCT03053050); and (2) compensated cirrhosis (STELLAR-4; NCT03053063) [8]. Furthermore, selonsertib is also being studied alongside and/or in combination with an acetyl-coenzyme A carboxylase inhibitor called GS-0976, and GS-9674 in a phase 2 clinical trial (ATLAS, NCT03449446) [65]. The primary outcome being measured is the proportion of patients experiencing adverse events, laboratory abnormalities, or greater than one stage of improvement in NAS without worsening of NASH at week 48 [65]. In a proof-of-concept study involving 70 subjects with NASH, selonsertib was studied in combination with GS-0976, and GS-9674, particularly with regard to changes in hepatic proton density fat fraction or liver stiffness [66]. At 12 weeks, patients treated with combinations of selonsetib with GS-0976 and selonsertib with GS-9674 demonstrated acceptable tolerability and improvements in hepatic de novo lipogenesis, liver biochemistries, and markers of fibrosis [65,66]. The combination of selonsertib and GS-0976, in particular, led to significant reductions in proton density fat fraction, alanine aminotransferase, and N-terminal propeptide of type III collagen [66]. The combination of selonsertib and GS-9674 led to reduction in gamma-glutamyl transpeptidase [66]. Based on these findings and pre-clinical data from the roles of GS-0976 and GS-9674 alone, such combinations were entered into a larger phase 2b study to evaluate patients with advanced fibrosis in the setting of NASH [67].

### 4.8. GS-0976: Acentyl-Coenzyme A Carboxylase Inhibitor

More recently, preliminary studies have evaluated the role of GS-0976 independently [68,69,70]. GS-0976 is an acetyl-coenzyme A carboxylase inhibitor, therefore inhibiting catalysis of de novo lipogenesis [68]. In a proof of concept, open-label study, treatment of 10 subjects with NASH with GS-0976 at 12 weeks resulted in decrease in hepatic de novo lipogenesis, proton density fat fraction, liver stiffness, and serum alanine transaminase, accompanied by favorable safety profile [68,69,70]. A separate phase 2 randomized, double-blind, placebo controlled trial evaluating 127 patients with NASH is in progress (NCT 02856555) [71].

### 4.9. SGM-1019: Inflammasome Inhibitor

Inflammasomes are activated in association with hepatocyte injury [10]. The inhibition of inflammasomes via SGM-1019 is being studied in preclinical and first-inhuman settings [10]. SGM-1019 was tested in rodents fed with a high-fat diet and carbon tetrachloride models of liver fibrosis in primates [10]. Its safety and pharmacologic activity was also assessed in healthy human volunteers [10]. SGM-1019 exhibited reduction of liver fibrosis scores in rodents as well as improvements in fibrosis, hepatocyte degradation and inflammation in the primates [10]. It was also well tolerated with a favorable safety profile as exhibited in humans over 2 weeks, prompting initiation of a phase 2a clinical trial [10].

### 4.10. Simtuzumab: Anti-LOXL2 Antibody

LOXL2 catalyzes cross-linkage of collagen causing remodeling of the extracellular matrix, which is a central phenomenon in fibrosis [11]. Murine studies showed that simtuzumab, a monoclonal antibody against LOXL2, may have an additive effect when combined with an ASK1 inhibitor, although additive efficacy was not demonstrated in combination with selonsertib as discussed in the aforementioned phase 2 clinical trial [8]. In the phase 2b, dose-ranging, randomized, placebo-controlled trial (NCT01672879), simtuzumab was tested in patients with NASH and bridging fibrosis or compensated cirrhosis [11]. Although well tolerated, simtuzumab demonstrated no efficacy as monotherapy, resulting in cessation of further studies [11].

### 4.11. JKB-121: TLR4 Antagonism

TLR4 has been hypothesized as a key mediator in innate immunity by triggering inflammatory responses through activation of genes encoding cytokines, chemokines, and antimicrobial agents [72]. It is expressed by Kupffer cells, hepatic stellate cells, biliary epithelial cells, hepatocytes, and liver sinusoidal endothelial cells [72]. TLR4 is upregulated in injured liver and induces inflammatory signaling cascades through lipopolysaccharide activation [72]. TLR4 has also been suggested as a player in hepatic fibrogenesis and an activator of macrophage recruitment to fibrogenesis sites [72]. Its antagonism by JKB-121 in animal models support reductions in lipopolysaccharide-induced inflammatory hepatic injury, cytokines IL6 and IL2, aminotransferase levels, mRNA expression of collagen, and hepatic stellate activation and proliferation, all of which have been noted for their involvement in the pathogenesis of NASH [73]. In a randomized, double-blind, placebo-controlled, parallel group phase 2a clinical trial (NCT02442687) in 65 patients with NASH, JKB-121, however, did not demonstrate superiority to the placebo at 24 weeks and instead revealed adverse events, two of which were severe [73,74]. It is unclear whether JKB-121 will proceed further beyond phase 2 clinical trials [75].

### 4.12. Additional Drugs in Phase 2a

**Figure 1:**
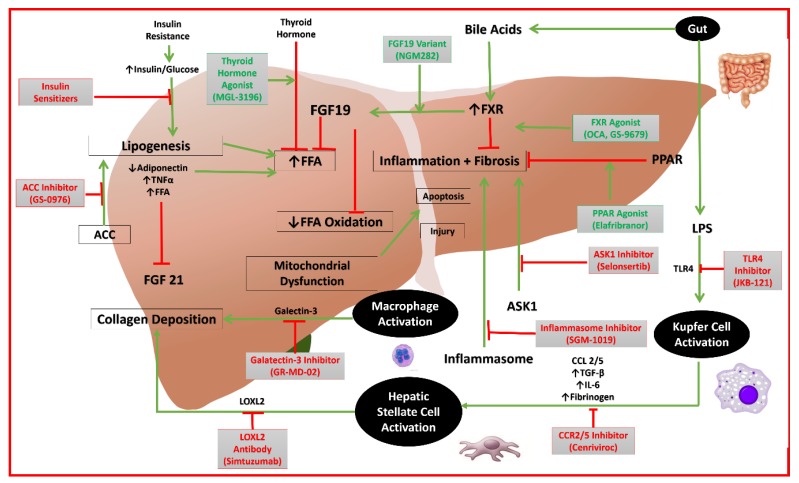
Mechanisms of action of emerging drugs for NAFLD. (FFA: free fatty acid; FGF19: fibroblast growth factor 19; PPAR: peroxisome proliferator-activated receptor alpha; TNF: tumor necrosis factor; TGF: transforming growth factor; IL: interleukin; LPS: lipopolysaccharide; ACC: acetyl-coenzyme A carboxylase; CCL: C-C chemokine ligand; CCR: C-C chemokine receptor; TLR: toll-like receptor; LOXL: lysl oxidase-like; ASK: apoptosis signal-regulating kinase; FXR: farnesoid X receptor).

**Figure 2:**
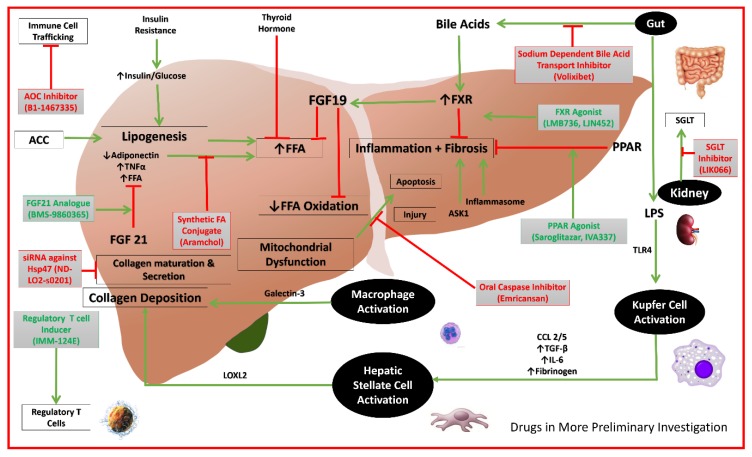
Mechanisms of actions of drugs in more preliminary investigations. (AOC: Amino-oxidase copper; FA: fatty acid; FFA: free fatty acid; FGF19: fibroblast growth factor 19; PPAR: peroxisome proliferator-activated receptor alpha; TNF: tumor necrosis factor; TGF: transforming growth factor; IL: interleukin; LPS: lipopolysaccharide; ACC: acetyl-coenzyme A carboxylase; CCL: C-C chemokine ligand; CCR: C-C chemokine receptor; TLR: toll-like receptor; LOXL: lysl oxidase-like; ASK: apoptosis signal-regulating kinase; FXR: farnesoid X receptor; SGLT: sodium-glucose cotransporter; siRNA: small interfering RNA; Hsp47: heat shock protein 47).

Other emerging drugs, for which there is minimal published data, can be found in Table 1 alongside the aforementioned therapies, phases, and mechanisms of action [76,77]. The mechanisms of action of the potential targets and their respective drugs are depicted in Figure 1 and Figure 2.

## 5. Future Directions

In this section we will briefly review emerging therapeutic and diagnostic tools. While several herbal agents may qualify as potential therapeutic candidates for patients with NAFLD, one agent deserves a mention here—shell ginger, a component in Japanese cuisine. The therapeutic mechanism of shell ginger resides in its highly potent action to scavenge free radicals which mediate obesity-related hepatocyte injury [78]. Therefore, shell ginger has been hypothesized to contribute to longevity by counteracting inflammatory disease processes, including NAFLD/NASH [78]. Hence, the tradition in Japanese population of consuming large amounts of shell ginger in their regular diet may have a protective effect against medical ailments triggered by free radical-based pathogenesis [78].

Among the promising diagnostic modalities on the horizon, the Roussel Uclaf Causality Assessment Method (RUCAM) has emerged as a reliable method for distinguishing idiosyncratic drug-induced liver injury (DILI) from NAFLD-related hepatic injury [79]. The RUCAM can be instrumental in determining the etiology of underlying liver injury [79,80]. The RUCAM is a scoring system which facilitates the diagnosis of DILI by utilizing the time to onset, risk factors, concomitant drugs, nondrug causes of liver injury, known hepatotoxicity of a drug, and response to re-challenge [79,80]. Prospective studies have not only highlighted the utility of the RUCAM in establishing a diagnosis of DILI but have allowed the identification of alternative causes of liver injury, including hepatitis E, hepatitis A, hepatitis B, autoimmune hepatitis, and sarcoidosis [80]. Thus, RUCAM may help in establishing the diagnosis of NAFLD, although future studies are warranted.

## 6. Conclusions

Current treatment guidelines support the notion of prevention and pre-emptive management of NAFLD by modification of modifiable metabolic and other risk factors. To date, pharmacotherapeutic agents are investigative and experimental. Therefore, extreme caution is warranted in using these experimental drugs without proper oversight. A majority of these novel and potentially effective agents/molecules/drugs should be used in the context of a clinical trial. These experimental drugs, including those proceeding through advanced phases of clinical trials and those that are not, may provide insight into the dominant pathogenetic pathways leading progressive liver injury associated with NASH [81].

## Figures and Tables

**Table 1:** Emerging drugs in the treatment of nonalcoholic fatty liver disease (NAFLD). FXR: farnesoid X receptor; PPAR: peroxisome proliferator-activated receptor; CCR: C-C chemokine receptor; FGF: fibroblast growth factor; TLR: toll-like receptor; NCT: National Clinical Trial.

	Drug Therapy	Current Clinical Trial Name	Phase	Mechanism of Action
**1**	**Obeticholic Acid**	REGENERATE; NCT02548351 [21]	3	FXR agonist
**2**	**GS-9674**	NCT02854605 [23]	2	FXR agonist
**3**	**Elafibranor**	RESOLVE-IT, NCT02704403 [28]	3	Dual PPAR α/δ agonist
**4**	**Cencriviroc**	AURORA; NCT03028740 [36,37]	3	CCR2 and CCR5 dual antagonist
**5**	**GR-MD-02**	NASH-CX [42,43]	2b	Galectin-3 inhibitor
**6**	**NGM282**	NCT02443116 [50]	2	Variant of FGF19
**7**	**MGL-3196**	NDT02912260 [53]	2	Selective thyroid hormone receptor-beta agonist
**8**	**Selonsertib**	STELLAR-3; NCT03053050 [8] and STELLAR-4; NCT03053063 [8]	3	Apoptosis signal-regulating kinase 1 inhibitor
**9**	**Simtuzumab**	NCT02466516 [62] and NCT01672879 [11]	2, 2b	Monoclonal antibody against LOXL2
**10**	**Selonsertib + Simtuzumab**	NCT02466516 [62]	2	
**11**	**GS-0976**	NCT 02856555 [70]	2	Acetyl-coenzyme A carboxylase (ACC) inhibitor
**12**	**Selonsertib + GS-9674**	ATLAS, NCT03449446 [64]	2	
**13**	**Selonsertib + GS-0976**	ATLAS, NCT03449446 [64]	2	
**14**	**JKB-121**	NCT02442687 [71]	2a	TLR-4 antagonist
**15**	**SGM-1019**	NCT	2a	Inflammasome inhibitor
**16**	**BMS-986036**		2a	Pegylated fibroblast growth factor 21 (FGF21) analogue [76,77]
**17**	**Aramchol**		2b	Synthetic fatty acid/bile acid conjugate [76,77]
**18**	**Volixibet**		2a	Apical sodium dependent bile acid transporter inhibitor [76,77]
**19**	**LMB763**		2a	FXR agonist [76,77]
**20**	**LJN452**		2a	FXR agonist [76,77]
**21**	**Emricasan**		2b	Oral caspase inhibitor [76,77]
**22**	**Saroglitazar**		2a	PPAR α/δ agonist [76,77]
**23**	**IVA337**		2a	Pan-PPAR agonist [76,77]
**24**	**IMM-124E-Hyperimmune bovine colostrum**		2a	Induction of regulatory T-cells [76,77]
**25**	**BI-1467335**		2a	Amino-oxidase copper (AOC) containing-3 inhibitor [76,77]
**26**	**LIK066**		2a	Sodium glucose cotransporter inhibitor (SGLT) [77]
